# Prevalence and physicians’ perception of workplace violence preventive measures in two Egyptian public hospitals

**DOI:** 10.1186/s42506-026-00213-6

**Published:** 2026-03-30

**Authors:** Mai E. El-Shishtawy, Wafaa W. Guirguis, Gehan R. Zaki, Taghareed A. Elhoseny

**Affiliations:** 1 Department of Health Administration and Behavioral Sciences, High Institute of Public Health, University of Alexandria, Alexandria, Egypt; 2 Department of Environmental Health, High Institute of Public Health, University of Alexandria, Alexandria, Egypt

**Keywords:** Workplace violence, Physicians, Hospital safety, Violence prevention, Occupational safety

## Abstract

**Background:**

Type II Workplace violence (WPV), in which the healthcare staff is attacked by the patients or their relatives/friends, is a significant issue in healthcare, impacting healthcare quality, worker safety, and organizational performance. Although studied frequently, further studies are needed in non-university hospital settings and departments other than the emergency. This study aimed to estimate the prevalence of physical and verbal type II WPV against physicians, identify the circumstances of the last incidents, and evaluate existing preventive measures from the physicians’ perspective.

**Methods:**

A descriptive cross-sectional design was used, recruiting a convenience sample of 324 physicians from one Health Insurance Organization (HIO) and one Ministry of Health and Population (MOHP) hospital in Alexandria (183 and 141 physicians, respectively). Data collection took place between August and November 2023 using a structured pre-validated self-administered questionnaire in English. The Statistical Package for Social Sciences (SPSS) version 25 was used for data analysis.

**Results:**

Verbal and physical WPV were reported by 61.4% and 8.3% of participants, respectively. Exposure was significantly associated with age group (30–40), single physicians, and residents’ position (*p* < 0.05). The patient’s relatives were the main aggressors. Physical violence was more common during the evening, while verbal violence was frequent in the morning. Emergency departments (EDs) were the primary sites of violence. Prevalent triggers included refusal of treatment, patient conditions, work overload, and long waiting times. Most incidents went unreported due to negligence and ignorance of reporting procedures. The HIO hospital showed better security measures, workplace procedures, staff training, and organizational support, while the MOHP hospital had better adequacy of staffing and resources.

**Conclusion:**

WPV is highly prevalent among the studied physicians, particularly in emergency settings and among junior staff. Institutional adoption of OSHA-based WPV prevention protocols remains inadequate, highlighting the need for systemic reform.

## Introduction

WPV has been addressed as a global problem, especially among healthcare workers (HCWs). It is defined as incidents where staff are abused, threatened, or assaulted in circumstances related to their work, including commuting to and from work, and involving an explicit or implicit challenge to their safety, well-being, or health [1]. WPV can be classified into four types: type I where the perpetrator is an outsider to the health care organization, type II where the perpetrator is either a patient or his family members/visitors, type III, in which the co-workers or supervisors are the perpetrator, or type IV, when the worker and perpetrator are personal/domestic partners, e.g., spouses. In this study, type II is the main concern. A large proportion of HCWs are occupationally exposed to type II WPV at work [2, 3]. Subtypes of type II WPV are verbal abuse (e.g., verbal threats, cursing, or shouting) and physical violence (e.g., hair pulling, slapping, hitting, or pushing) [1, 4].

A systematic review and meta-analysis of 78 studies published between 1987 and 2018 reporting on the pooled prevalence of WPV among HCWs worldwide estimated that 61.9% of HCWs were exposed to one or more WPV incidents during the previous year, the figure ranging from 48.1% to 70.9% and that nurses had the highest exposure to any form of WPV, followed by physicians and other healthcare professionals (59.2% vs. 56.8% vs. 44.4%, respectively) [3]. A systematic review and meta-analysis of WPV among physicians, based on 6 studies published between 2012 and 2015, reported 69% as the pooled 1-year prevalence, with the highest prevalence (86%) in Turkey, and the lowest one (54%) in Germany [5]. A systematic review of 22 peer-reviewed studies on WPV in Africa revealed a prevalence ranging from 9% to almost 100%, with the highest rates reported from South Africa and Egypt [6]. In Egypt, the prevalence of WPV among physicians was reported to be high, with the highest 1-year prevalence (92.6%) in the Emergency Hospital of Mansoura University, and the lowest (56.40%) in Tanta hospitals [4, 7–14].

There are five main categories of WPV determinants: patient-related factors, physician-related factors, worksite design-related factors, organizational factors, and societal factors [15]. It was found that patients who committed WPV were more likely to be males, from rural areas, with altered mental status, in pain, with a low level of life satisfaction, dissatisfied with the quality of care, and with high income [16–18]. Physicians who were younger, less experienced, lacked communication skills, and worked in surgical specialties, emergency departments (EDs), and psychiatric hospitals were more exposed to WPV [12, 18, 19]. Worksite design-related factors include a lack of controls in accessing staff-only and patient areas, uncomfortable waiting areas, and poor lighting [15]. On the organizational level, HCWs were more vulnerable to WPV in organizations that considered WPV as a part of the job, had supply and staff shortages with an increased workload, and did not provide training courses on WPV [17, 19, 20]. Society-related factors include the prevalence of poverty, unemployment, and weapons in areas served by the hospital, and hostile patient-provider interactions triggered by the mass media [15, 21].

Physicians’ responses to WPV varied; some preferred to take no action, while others replied verbally or physically, left the scene, obtained psychological support, got training courses on WPV, reported to the supervisors or the manager, called the police, asked to change their position, or left the hospital [22–24]. Most studies revealed that physicians are under-reporting violence incidents. The reasons for under-reporting included a lack of reporting system privacy or its complexity, considering violence as a part of the job nature, and fear of consequences [17, 25]. The consequences of WPV affect the violated physicians as well as the organization [3]. For physicians, the most frequent outcome was psychological, e.g., depression, low self-esteem, feeling unsafe, and self-blaming [17, 18, 26, 27] in addition to physical injuries or death [26, 28]. Consequences for the organization include staff burnout and turnover, lower job performance, and dissatisfaction among physicians [12, 29, 30].

In view of the considerable impact of WPV, there is a consensus that violence prevention plans should be established and implemented. The Occupational Safety and Health Administration (OSHA) recommends an integrated WPV prevention program consisting of five elements, namely, management commitment and employee participation, worksite analysis, hazard prevention and control, safety and health training, and recordkeeping and program evaluation [26]. Some preventive measures include environmental controls (e.g., adequate security, good lighting and comfortable waiting areas), workplace controls (e.g., adequate staff number, availability of equipment, and training courses on violence), administrative controls (e.g., increasing the authority of physicians and announcing non-tolerance for WPV [29, 31], as well as organizational support (such as rapid action from the manager, encouraging reporting, and analyzing the event to detect risks) [15, 26]. In Egypt, the General Authority for Healthcare Accreditation and Regulation (GAHAR) standards for hospitals emphasize the importance of risk assessment and prevention, positive organizational culture, and a safe environment to prevent violence [32].

Most research in Egypt was conducted in university hospitals and focused on the emergency department [7–10, 14]. As information is scarce about other types of hospitals and other departments, the current study focused on type II WPV among physicians in all departments of two hospitals affiliated to the Health Insurance Organization (HIO) and the Ministry of Health and Population (MOHP). The objectives of the present study were to estimate the prevalence of type II WPV (physical and verbal violence) against physicians, to determine its circumstances, and to identify the preventive measures applied by the study hospitals to combat WPV, from the physicians’ point of view.

## Methods

### Study design and settings

A descriptive cross-sectional design was used.

This study was conducted in two general hospitals in Alexandria, Egypt; one is the largest MOHP hospital in terms of number of beds, and the second is the largest hospital affiliated with the HIO.

### Study populations and sampling techniques

The study population included physicians working in the study hospitals. Sample size was calculated using Epi Info version 7. With the assumption that 50% of physicians working in each hospital would be exposed to WPV during the last 12 months, to achieve a 95% confidence level around the expected prevalence with 5% precision, and based on a population size of 349 physicians in the HIO hospital and 220 physicians in the MOHP hospital, the minimum required sample size was 183 from the HIO hospital and 141 physicians from the MOHP hospital.

### Type of sample and method of selection

A convenience sampling technique was used as the questionnaire was distributed to all physicians working in the study hospitals, and returned questionnaires were collected daily until the required sample size was reached. A convenience sampling technique was followed to ensure voluntary participation.

### Data collection methods and tools

A structured pre-validated self-administered questionnaire was administered to the study sample in English. It was formed of three sections.

*The first section* covered the sociodemographic characteristics of physicians, such as age, gender, and the occupational characteristics of physicians, such as position, specialty, department, and years of experience in the study hospital.

*The second section* was adapted mainly from the WPV in the health sector survey tool developed through collaborations between the World Health Organization, International Labour Office, International Council of Nurses, and Public Services International [33] to collect information about the rate of occurrence of and witnessing WPV. Details about the last incident of physical and verbal violence in the study hospitals during the previous year were investigated, including type, circumstances, actions taken by physicians exposed to violence, reporting, and consequences of WPV exposure on the physicians (psychological or physical), on their job and on patient care.

*The third section*** is** adapted mainly from the OSHA guidelines [26] and other relevant studies [13, 15, 31]. assessed physicians’ perception of WPV preventive measures in both hospitals. This section included the occupational-related risk factors, the existing workplace design and environmental control measures, and organizational preventive measures **(**staffing and resources, security measures, workplace procedures, training, and organizational support). A 3-point Likert scale (yes, no, or do not know) was used.

For the existing preventive measures in the hospitals studied, percentage scores were calculated from the 3-point scale (yes = 2, do not know = 1, and no = 0). The percentage score of each category was calculated using the following equation:

$$\mathrm{Percentage}\:\mathrm{score}\:\mathrm{of}\:\mathrm{each}\:\mathrm{category}=100\times\frac{\mathrm{Total}\:\mathrm{score}\:\mathrm{of}\:\mathrm{participants}^\prime\:\mathrm{responses}}{\mathrm{Number}\:\mathrm{of}\:\mathrm{questions}\times2}$$

the percentage score of physicians’ perception of the existing preventive measures to combat WPV (%PM) was rated as good (≥ 80%), moderate (60%−79%), and poor prevention perception level (< 60%).

Face validity and content validity of the data collection tool were examined by three experts. A pilot study was conducted in July 2023, among a sample of ten physicians. The sample from the pilot study was not included in the analysis. Based on their feedback, confusing and ambiguous questions were removed, and some modifications were made to improve the final version of the questionnaire. It revealed that the time required to answer the questionnaire was 20 to 30 min. The questionnaire collected data on occurrences of violence incidents and their characteristics, with no perceptions, so a reliability check was not needed. Data were collected from August 2023 to November 2023.

### Statistical analysis

Data were entered, coded, and statistically analyzed using the Statistical Package for Social Sciences (SPSS) version 25. The Kolmogorov–Smirnov test was used to test the normality of quantitative variables. Chi-square or Fisher’s exact tests were used for categorical variables as appropriate. The Mann-Whitney U test was applied for non-normally distributed continuous variables. A p-value < 0.05 was considered statistically significant.

Abnormally distributed quantitative data were presented using median, minimum, and maximum. Mann-Whitney U test was applied to determine the association between the abnormally distributed percentage scores of the occupational risk factors and the existing preventive measures, with the type of the hospital.

## Results

A total of 82.7% of physicians experienced WPV, with verbal abuse being the predominant form. Notably, 61.7% reported verbal violence in the past year, mostly episodic, while physical violence was reported by 8.3%. Nearly all (95.1%) witnessed WPV before, 95.4% witnessed verbal violence, and 40% witnessed physical violence during the past year. The vast majority of the sample (95.4%) were worried about WPV exposure (Table 1).

**Table 1:** Distribution of the study sample of physicians according to exposure to or witnessing WPV (Alexandria, 2023)

Variable	No. (*n*=324)	%
Ever Exposure to violence		
yes	268	82.7
no	56	17.3
Type of violence	(n=268)	
verbal only	216	80.6
physical only	0	0.0
both verbal &physical	52	19.4
Past year exposure		
Verbal violence		
yes	200	61.7
no	124	38.3
Frequency	(n=200)	
daily	8	4.0
1–3 times/week	23	11.5
1–3 times/month	77	38.5
1–3 times/year	92	46.0
Physical violence		
yes	27	8.3
no	297	91.7
Frequency	(n=27)	
daily	0	0.0
1–3 times/week	0	0.0
1–3 times/month	3	11.1
1–3 times/year	24	88.9
Ever witness		
yes	308	95.1
no	16	4.9
Past year witness		
Verbal violence		
yes	309	95.4
no	15	4.6
Physical violence		
yes	130	40.1
no	194	59.9
Worry about exposure		
yes	327	95.4
no	15	4.6

About four fifths of physicians (79.6%) were 40 years old or younger. About half of them were males (50.3%) and single (51.9%). The highest percentage was residents (58.9%), had 10 years or less of work experience (62.7%) and belonged to a medical specialty (47.5%). There was a statistically significant association between younger age, being single, and the resident position and exposure to verbal violence (*p* = 0.009, 0.002, and < 0.001, respectively). Also, being single and having a lower number of work experience years had a significant statistical association with physical violence exposure (*p* = 0.008 and 0.038, respectively) (Table 2).

**Table 2:** Distribution of physicians according to socio-demographic and occupational characteristics and exposure to violence during the last year (Alexandria, 2023)

Characteristic	Total *N*=324		Verbal violence exposure					Physical violence exposure				
			Yes (*n*=200)		No (*n*=124)		*p*-value	Yes (*n*=27)		No (*n*=297)		*p*-value
	No.	%	No.	%	No.	%		No.	%	No.	%	
Socio-demographic												
Age												
<30	108	33.3	71	35.5	37	29.8	0.009*	14	51.9	94	31.6	0.176†
30–40	150	46.3	96	48.0	54	43.5		12	44.4	138	46.5	
>40–50	35	10.8	23	11.5	12	9.7		1	3.7	34	11.4	
>50–60	25	7.7	9	4.5	16	12.9		0	0.0	25	8.4	
>60	6	1.9	1	0.5	5	4.0		0	0.0	6	2.0	
Gender												
Male	163	50.3	108	54.0	55	44.4	0.110	13	48.1	150	50.5	0.815
Female	161	49.7	92	46.0	69	55.6		14	51.9	147	49.5	
Marital status												
Single	168	51.9	114	57.0	54	43.5	0.022*	21	77.8	147	49.5	0.008*
married	156	48.1	86	43.0	70	56.5		6	22.2	150	50.5	
Occupational												
Position												
intern	8	2.5	5	2.5	3	2.4		0	0.0	8	2.7	0.131†
resident	191	58.9	125	62.5	66	53.2		21	77.8	170	57.2	
ass.specialist	37	11.4	25	12.5	12	9.7	<0.00*	4	14.8	33	11.1	
specialist	59	18.2	39	19.5	20	16.1		2	7.4	57	19.2	
consultant	29	9.0	6	3.0	23	18.5		0	0.0	29	9.8	
Experience												
<10 years	203	62.7	128	64.0	75	60.5	0.525	22	81.5	181	60.9	0.038*
>10 years	121	37.3	72	36	49	39.5		5	18.5	116	39.1	
Specialty												
Surgical	90	27.8	59	29.5	31	25.0		9	33.3	81	27.3	
Medical	154	47.5	91	45.5	63	50.8		15	55.6	139	46.8	0.263†
Obst/gyn	39	12	22	11.0	17	13.7	0.394	0	0.0	39	13.1	
ER	16	4.9	13	6.5	3	2.4		2	7.4	14	4.7	
ICU	25	7.7	15	7.5	10	8.1		1	3.7	24	8.1	

*ass. specialist* assistant specialist, *obst/gyn* obstetrics and gynecology

Chi-square test was used except where ^†^MC: Monte Carlo test was used

^*^Significant at P<0.05 at 95% confidence interval

The last verbal violence incident during the last year was most commonly in the form of shouting (93.5%) followed by foul words (34%) and verbal threats (12.5%) while the last physical violence incident was most commonly in the form of pushing (92.5%) followed by kicking (7.4%) and hitting (7.4%) In both verbal and physical violence, the perpetrator was mostly the relatives or visitors (76% and 88.9%, respectively). The last verbal violence incident occurred mostly during morning and evening shifts (41.5% and 38%, respectively), while the last physical violence incident occurred more frequently during evening and night shifts (48.2% and 29.6%, respectively). Regarding place, both verbal and physical incidents occurred mostly in the ER (49.5% and 70.4%, respectively) and the outpatient department (28.5% and 11.1%, respectively). Physicians were attacked while taking history or examination in 46.0% of verbal violence incidents and 37.0% of physical violence cases, and while taking care of another patient in 42.5% of verbal violence incidents and 44.4% of physical violence cases (Table 3).

**Table 3:** Characteristics of the last violent incident in the past year (Alexandria, 2023)

Characteristic	Verbal violence		Physical violence	
	No.(*n*=200)	%	No.(*n*=27)	%
Type^a^				
shouting	187	93.5	-	-
foul words	68	34.0	-	-
verbal threats	25	12.5	-	-
cursing	8	4.0	-	-
rude tones	6	3.0	-	-
pushing	-	-	25	92.5
kicking	-	-	2	7.4
hitting	-	-	2	7.4
scratching	-	-	1	3.7
Perpetrators				
patient	24	12.0	2	7.4
relatives/visitors	152	76.0	24	88.9
both	24	12.0	1	3.7
Shift				
morning	83	41.5	5	18.5
evening	76	38.0	13	48.2
night	37	18.0	18	29.6
don’t remember	4	2.0	1	3.8
Place				
ER	99	49.5	19	70.4
outpatient	57	28.5	3	11.1
wards	25	12.5	1	3.7
ICU	16	8.0	0	0.0
on stairs	2	1.0	1	3.7
the lift	1	0.5	3	11.1
Attacked during				
taking history or examination	92	46	10	37.0
taking care of another patient	85	42.5	12	44.4
patient admission	14	7.0	0	0.0
patient referral	4	2.0	1	3.7
patient discharge	1	0.5	0	0.0
during the visit	1	0.5	0	0.0
riding the lift	1	0.5	4	14.8
don’t remember	2	1.0	0	0.0

^a^More than one response allowed

The most common predisposing factors for verbal violence were refusal of hospital policies by patients (40.0%), workload (35.0%), long waiting time (34.5%) and patient condition (19.0%) while the most frequent predisposing factors for physical violence were workload (37.0%), long waiting time (37.0%), patient condition (33.3%) and refusal of hospital policies (29.6%). The most common consequence of exposure to both verbal and physical violence was decreased job performance and satisfaction (69% and 74.1%, respectively). The table shows that 97.5% of verbal violence and 88.9% of physical violence incidents were not investigated (Table 4).

**Table 4:** Predisposing factors and consequences of the last violent incident in the past year (Alexandria, 2023)

Characteristic	Verbal violence		Physical violence	
	No.(*n*=200)	%	No.(*n*=27)	%
Predisposing factor				
refusal of hospital policies by patient	80	40.0	8	29.6
workload	70	35.0	10	37.0
long waiting time	69	34.5	10	37.0
patient condition	38	19.0	9	33.3
unavailability of equipment	16	8.0	2	7.4
inadequate security	7	3.5	4	14.8
VIP patient or relative	3	1.5	0	0.0
lack of experience in dealing with violence	1	0.5	0	0.0
don’t remember	3	1.5	0	0.0
Consequences^a^				
decreased job performance and satisfaction	138	69.0	20	74.1
care of the patient discontinued	108	54.0	3	11.1
phycological distress	21	10.5	19	70.4
asked for a day off	12	6.0	0	0.0
injured	4	14.8	4	14.8
none	28	14.0	3	11.1
Incident investigation				
management or Police	5	2.5	3	11.1
none	195	97.5	24	88.9

^a^More than one response allowed

In response to the last verbal and physical violence incidents, most frequently, physicians called the security (55.5% and 81.5%, respectively) and replied verbally (50% and 37%, respectively). The vast majority of physicians did not file an administrative complaint after exposure to verbal violence (99%) or physical violence (88.9%). The most common reasons for not filing an administrative complaint after verbal and physical violence were negligence of previous violence complaints (38.9% and 58.3% respectively), the patient/relative apologized (35.9% and 25.0% respectively), and did not know how to report, or to whom (21.7% and 29.2% respectively). After exposure to the last verbal violent incident, 34.0% intended to quit, 75% of them intended to leave to another country; while 66.7% intended to quit after exposure to the last physical incident, 83% of them intended to leave to another country (Table 5).

**Table 5:** Physicians’ responses to the last violence incident in the past year (Alexandria, 2023)

Response	Verbal		Physical	
	No. (*n*=200)	%	No. (*n*=27)	%
Action taken^a^				
called the security	111	55.5	22	81.5
replied verbally	100	50.0	10	37.0
told the senior	18	9.0	0	0.0
told a colleague	17	8.5	1	3.7
left place of the incident	15	7.5	1	3.7
took no action	14	7.0	0	0
reported to management	7	3.5	2	7.4
told your family	6	3.0	1	3.7
called the police	3	1.5	4	14.8
defended himself physically	2	1.0	2	7.4
Reporting				
Yes	2	1.0	3	11.1
No	198	99.0	24	88.9
Reason for non-reporting^a^	(n=198)		(n=24)	
negligence of previous complaints	77	38.9	14	58.3
the patient/relative apologized	71	35.9	6	25.0
did not know how to report	43	21.7	7	29.2
consider violence as part of the job	38	19.2	4	16.7
it was not worth it	36	18.2	0	0.0
did not have time due to workload	30	15.2	2	8.3
avoiding troubles	16	8.1	1	4.2
called the police	2	1.0	4	16.7
afraid of negative consequences	1	0.5	1	4.2
lack of reporting privacy	0	0.0	1	4.2
Intention to quit				
Yes	68	34.0	18	66.7
No	132	66.0	9	33.3
Physicians’ options for quitting^a^	(n=68)		(n=18)	
go to another hospital	10	14.7	3	16.7
change specialty	5	7.4	1	5.6
go to another country	51	75.0	15	83.3
shift career	15	22.1	2	11.1

^a^More than one response allowed

Concerning preventive measures of WPV, the environmental measures and workplace designs according to physicians opinion it showed defects concerning availability of places for protection during violence, enough exits to escape in case of violence, restricted public access to reception, comfortable waiting areas in both hospitals and ability of physicians to observe patients in waiting areas in both hospitals with a significant higher positive responses from the HIO hospitals (30.1%, 32.8%, 32.8%, 35.5% and 39.9% respectively) compared to the MOHP hospital (4.9%, 0.7%, 4.3%, 7.8% and 17.0% respectively) (*p* < 0.001 for all). Enough lighting to see clearly, waiting areas free of objects that could be used as weapons, work areas free of objects that could be used as weapons, avoiding being trapped by arranged furniture in waiting areas and avoiding being trapped by arranged furniture in work areas were significantly higher in the MOHP hospital (89.4%, 96.5%, 89.4%, 84.4% and 98.6% respectively) compared to the HIO (77.6%, 52.5%, 54.7%, 63.4% and 59.0% respectively) (*p* < 0.001 for all). Concerning the staffing and resources, both hospitals had security staff inside and outside the hospital. Other measures such as physicians not working alone, suitable physicians’ numbers, knowing the security staff names and provision of suitable equipment for work were significantly more positively reported for the MOHP hospital (97.2%, 89.4%, 88.7% and 80.1% respectively) than the HIO hospital (66.7%, 58.5%, 63.4% and 85.5% respectively) (*p* < 0.001 for all). Regarding security measures, metal detectors and alarm systems were reported as positive only by 3.3% of the HIO hospital physicians. Concerning other security measures inside the MOHP hospital, including the locks of doors of the physician’s residence, internal phone system to call for help in case of violence, and the presence of a police station, were reported as positive by 0.0%, 5.0%, and 0.7%, respectively. The workplace procedures were deficient in both hospitals, except for wearing an Identification Document (ID), as reported by 91.5% of the MOHP hospital physicians (Table 6**).**

**Table 6:** Distribution of physicians’ positive responses for the existing measures to combat WPV according to hospital type (Alexandria, 2023)

Measure	HIO (*n* = 183)		MOHP (*n* = 141)		*P*-value
	No.	%	No.	%	
Design					
Enough lighting to see clearly	142	77.6	126	89.4	0.005^*^
Adequate ventilation	131	71.6	82	58.2	0.014^* †^
Private, locked bathrooms are available for staff	154	84.2	60	42.6	< 0.001^*†^
Availability of places for protection during violence	55	30.1	7	4.9	< 0.001^*^
Enough exits to escape in case of violence	60	32.8	1	0.7	< 0.001^*^
Different entries to reach the work areas	76	41.5	89	63.1	< 0.001^*^
Restricted public access to the reception	60	32.8	6	4.3	< 0.001^*^
Restricted public access to work areas	72	39.3	103	73.0	< 0.001^*†^
Someone can hear a physician’s call for help	179	97.8	122	86.5	< 0.001^*^
Comfortable waiting areas	65	35.5	11	7.8	< 0.001^*^
Waiting areas free of objects that could be used as weapons	96	52.5	136	96.5	< 0.001^*†^
Work areas free of objects that could be used as weapons	100	54.7	126	89.4	< 0.001^*^
Avoiding being trapped by arranged furniture in waiting areas	116	63.4	119	84.4	< 0.001^*^
Avoiding being trapped by arranged furniture in work areas	108	59.0	139	98.6	< 0.001^*^
Physicians can observe patients in waiting areas	73	39.9	24	17.0	< 0.001^*†^
The parking area is safe	103	56.3	24	17.0	< 0.001^*†^
Staffing and resources					
The physician is not working alone	122	66.7	137	97.2	< 0.001^*^
Suitable physicians’ numbers	107	58.5	126	89.4	< 0.001^*†^
Suitable workload	78	42.6	80	56.7	0.014^*^
Security staff are available inside the hospital	183	100	141	100	
Know the security staff names	116	63.4	125	88.7	0.001^*^
Healthcare staff can ask for security personnel easily and promptly	118	64.5	79	56.0	0.105^†^
Security personnel have sufficient authority	71	38.8	8	5.7	< 0.001^*†^
Security personnel are available outside the hospital	183	100	141	100	
Suitable equipment is provided for work	107	58.5	113	80.1	< 0.001^*†^
Security measures					
Security cameras in high-risk areas	79	43.2	62	44.0	< 0.001^*^
Alarm systems	6	3.3	0	0.0	0.019^*†^
Metal detectors	0	0.0	0	0.0	0.003^*^
Doors of the physician’s residence have locks	81	44.3	0	0.0	< 0.001^*^
Internal phone system to call for help in case of violence	134	73.2	7	5.0	< 0.001^*^
Police station inside the hospital	171	93.5	1	0.7	< 0.001^*^
Workplace procedures					
A crisis management plan is present	72	39.3	21	14.9	< 0.001^*^
The crisis management plan contains scenarios of possible violent incidents	7	3.8	0	0.0	0.003^*†^
A telephone number is posted in a clear area to use in case a crisis happens	11	6.0	0	0.0	0.001^*†^
Written work violence protocols and procedures are present	0	0.0	0	0.0	< 0.001^*^
A written program for WPV prevention is present	0	0.0	0	0.0	0.033^*^
Managers emphasize that violence should not be tolerated, and it is not a part of the job	28	15.3	1	0.7	< 0.001^*^
A reporting system for violent incidents is available	6	3.3	0	0.0	< 0.001^*†^
Notification of workers of past violent acts by particular patients	33	18.0	0	0.0	< 0.001^*†^
Short waiting times to prevent patients’ frustration	72	39.3	73	51.8	< 0.005^*†^
Warning signs for the public stating that violence is not accepted	0	0.0	0	0.0	0.002^*^
Floor plans are available	5	2.7	0	0.0	0.004^*†^
Broken windows and locks are repaired promptly	92	50.3	96	68.1	0.003^*^
Special security measures to protect people who work late at night	16	8.7	2	1.4	< 0.001^*^
Visitors are asked to wear ID badges	0	0.0	0	0.0	0.011^*†^
IDs are required for the staff	102	55.7	129	91.5	< 0.001^*^
Training					
Crisis response plan	5	2.7	11	7.8	0.066^†^
Reporting violent incidents or threats	2	1.1	0	0.0	0.508^†^
Handling aggressive patients	0	0.0	0	0.0	
Preventing potentially violent situations (de-escalating techniques)	2	1.1	0	0.0	^FE^*p*=0.507^£^
Personal safety and self-defense	3	1.6	0	0.0	^FE^*p*=0.260^£^
Communication skills	29	15.8	0	0.0	^FE^*p*=0.006^*£^
Security staff are adequately trained	29	15.8	1	0.7	< 0.001^*†^
Organizational support					
Rapid action of the manager in case of violence to support the victim	38	20.8	12	8.5	< 0.001^*^
There is psychological support from the manager to the victim	10	5.5	1	0.7	< 0.001^*^
The manager encourages reporting of violent incidents	26	14.2	1	0.7	< 0.001^*^
An analysis of the violent incident is done to know the causes	11	6	2	1.4	< 0.001^*^

Chi-square test was used except where ^†^*MC* Monte Carlo test was used or £ Fisher’s exact test was used

^*^Significant at *P* < 0.05 at 95% confidence interval

No significant difference was found regarding the environmental and workplace design preventive measures. However, Table 7 shows significant differences in all organizational-related preventive measures between the HIO and MOHP hospitals. Regarding the staffing and resources-related preventive measures, both hospitals had moderate levels of prevention. The HIO hospital had a significantly lower %PM (66.7% with a range of 22.2%–100.0%) than that of the MOHP hospital (77.8% with a range of 33.3%–100.0%) (U = 9739.5, *P* < 0.001). As regards security and workplace preventive procedures, both hospitals had poor levels of prevention. There is a significantly higher security and workplace %PM at the HIO hospital (50.0% and 30.0%) with ranges of 8.3%–91.7% and 3.3%–73.3%, respectively, compared to the MOHP’s medians of 16.7% (range 0.0%–33.3%) and 26.7% (range 10.0%–46.7%), respectively (U = 9685.0 and 11073.0, respectively, and *P* < 0.001 for each). Similarly, for the organizational support, the HIO hospital had a significantly higher median (25.0%) with a range of 0.0%–100.0% compared to that of MOHP’s (12.5%) with a range of 0.0%–87.5%, with both hospitals recording poor levels of organizational support (U = 933.0, *P* < 0.001). Finally, poor levels of provided training were found for both hospitals. The HIO hospital had a significantly higher maximum percentage score of 42.9% than that of the MOHP hospital (14.3%), with median scores and minimum scores of 0.0 for each (U = 11815.5, *P* = 0.001).

**Table 7:** Percentage score of physicians’ perception of the preventive measures of WPV according to type of hospital (Alexandria, 2023)

Variable	%PM		*p*-value
	HIO(*n* = 183)	MOHP(*n* = 141)	
1. Environmental and workplace design			
Median	56.3	56.3	0.167
Min-max	12.5–93.8	28.1–75.0	
Rating of prevention level	Poor	Poor	
2. Organization-related preventive factors			
• Staffing & resources			
Median	66.7	77.8	<0.001*
min-max	22.2–100.0	33.3–100.0	
Rating of prevention level	Moderate	Moderate	
• Security preventive measures			
Median	50.0	16.7	<0.001*
min-max	8.3–91.7	0.0–33.3	
- Rating of prevention level	Poor	Poor	
• Workplace preventive procedures			
Median	30.0	26.7	<0.001*
min-max	3.3–73.3	10.0–46.7	
Rating of prevention level	Poor	Poor	
• Training			
Median	0.0	0.0	0.001*
min-max	0.0–42.9	0.0–14.3	
Rating of prevention level	Poor	Poor	
• Organizational support			
Median	25.0	12.5	<0.001*
min-max	0.0–100.0.0.0	0.0–87.5.0.5	
Rating of prevention level	Poor	Poor	

The Mann-Whitney U test is used

*HIO* Health Insurance Organization, *MOHP* Ministry of Health and Population, *%PM* Percentage score of physicians’ perception of the existing preventive measures to combat violence

^*^Significant at *P*<0.05 at 95% confidence interval

## Discussion

WPV has become a major concern in healthcare in Egypt. Physicians, being the team leaders within healthcare encounters, represent the main victims as targets of frustration and aggressive behaviour of patients and their companions. There is an escalating pattern of this problem [33], which has driven a significant proportion of physicians to consider leaving the country. The untreated root causes of this outside movement of physicians aggravate the shortage of physicians experienced nowadays [33–35].

This study showed that healthcare sectors other than the university sector are similarly exposed to the dangers of WPV. It was found that 82.7% of physicians were exposed to violence. This rate is remarkably high when compared to a global prevalence of 63.1% of WPV in healthcare settings of any type [36]. The past year exposure is comparable to that reported by a systematic review and meta-analysis of 253 eligible studies including 331,544 participants. This study showed a 61.9% reported rate of exposure to WPV of any form in the past year [3]. The current system is to distribute emergency cases by ambulance between the city hospitals, including MOHP hospitals, and not only university hospitals as before. These hospitals are now receiving emergency cases and are subject to the pressure of workload, with the possible deficiency of specialized staff and resources. Health insurance hospitals also receive large numbers of outpatient visits who need treatment, medicine, surgical operations, and approval for sick leaves. These insurers are subjected to the pressure of long waiting times and deficient staff and resources. These circumstances provoke violent behaviour towards the working staff.

Egyptian studies of WPV reported a similar rate in many governorates, such as Assiut (78.2%), Beni-Suif (80.3%), and Alexandria (91.7%) [4, 8, 11]. Verbal violence was more commonly occurring than physical violence, and this is reported by other studies on WPV [37, 38]. Similarly, a study in Egypt that involved 250 physicians from 13 Egyptian governorates reported that verbal violence is more prevalent than physical violence (88% and 42%, respectively) [39].

The age group 30–40 years and residents were more exposed to verbal violence, and working experience of less than 10 years is significantly associated with physical violence. This may be due to the presence of residents and younger physicians in direct contact with patients and their families, which makes them more likely to be exposed to their reactions. Many studies reported that younger age [3, 37, 40, 41] is significantly associated with exposure to WPV, and junior residents were found to be more exposed to WPV than senior residents and consultants [3, 37]. Meanwhile, other studies showed that older age [42] and longer experienced physicians are more frequently exposed to WPV [39, 42], while others showed no correlation between age, gender, work experience, marital status, hierarchy, and WPV [43–45]. This can draw attention to the wide-scale occurrence of WPV among physicians of different characteristics. This necessitates the presence of a comprehensive preventive program that covers all physicians regardless of age, sex, experience, or other characteristics. Healthcare workers need to be protected by laws, administrative regulations, and special training on how to deal with violence and its provoking factors.

The current study shows that the majority of physicians were exposed to verbal violence in the form of shouting and foul words, while pushing was the most common form of physical violence. This is similar to other studies in Egypt. It was shown that pushing or pulling and imposing harm by hands were the most common forms of physical violence [39, 43] and shouting was the most prevalent form of verbal violence [43, 45]. The perpetrator was a member of the patient’s family or friends (67.0% for verbal and 88.9% for physical incidents), which is similar to other studies [2, 8, 39]. Patients being weak due to their health status are not part of the violent attacks. In Egypt, traditionally, patients are accompanied by a large number of family members and friends as a sort of solidarity in difficult times. In this case, policies that regulate the allowed number of companions should be issued and imposed. In addition, security staff who are trained in how to deal with them during stressful situations should be in adequate numbers and be well-trained to detect signs of possible violence.

Verbal violence occurred mostly during morning and evening shifts nearly equally (41.5% for the former and 38.0% for the latter) while physical violence was most common during evening shifts (48.2%) followed by night shifts (29.6%). Morning and evening shifts always receive the highest number of patients, bringing a high workload and long waiting times. It is worth mentioning that morning shifts are the time of outpatient clinics where a considerable percentage of verbal incidents took place (28.5%), while evening shifts are the periods of the day when visitors are allowed. On the other hand, the night shifts, when about one-third of physical incidents occurred, are characterized by anxious attendants reaching the ED with acute cases seeking urgent help and in imminent fear of serious outcomes. Furthermore, this shift is mostly staffed with junior medical personnel, and senior staff are frequently unavailable. WPV was reported to occur during morning shifts [13], but in emergency departments, it is more common to be in the evening [14] and on night shifts [39, 43, 46]. The emergency department is the most commonly reported site of WPV in hospitals [3, 8, 13, 45]. In the present study, all the hospital departments were included in the survey, but still ED was the most common place of violence.

Both verbal and physical violence occurred mostly during physician contact with the patients, either during history taking, patient examination or taking care of another patient. From the physicians’ point of view, the predisposing factors for violence are high workload, long waiting time, and poor patient condition. Refusal of hospital policies such as those for admission or discharge, unavailability of equipment, and inadequate security were mentioned, too. None of these factors pointed accusation to the patients. In another study in a university hospital in Alexandria, physicians gave similar reasons for patients’ violence, including the shortage of staff, high workload, overcrowding, shortage of resources, and poor management, in addition to patient-related factors as poor attitude and lack of awareness [8]. In the current study, although none of the staff mentioned patient-related factors except their health state, some violent incidents took place away from the patients’ encounters as those in the hospital elevators.

These results are similar to other studies measuring WPV-related factors in or out of Egypt. Service-related factors, such as inadequate security, shortage of resources, high workload, overcrowding, and inadequate experience of the staff, were reported [13, 39, 43]. Others mentioned patient-related factors, such as low socioeconomic status and low education level of patients, drug abuse by patients, and psychiatric disorders [13, 38, 43]. Provision of adequate resources to reduce the long waiting time is expected to reduce patients’ and families’ anger. Mass media involvement and the spread of public awareness of the right public behaviour within the healthcare facilities are beneficial.

In the current study, 74.1% and 69% of physicians exposed to physical and verbal violence, respectively, reported reduced job performance and satisfaction, and discontinuation of the patient care process after verbal violence. WPV impacts the well-being of medical staff and the process of provision of medical care either directly through interference and interruption of work or indirectly, as medical staff will not practice normally after violence exposure, and many of them consider quitting.

In response to violence, the majority of physicians did not file an administrative complaint. They mostly called the security or replied verbally. Negligence of previous violence complaints was the most common cause of non-filing of a complaint. As the administration did not help physicians and did not give them a response when notified of the incidents, this made physicians feel that it was not worth notifying. Under-reporting was noticed despite the high rate of exposure. Cultural normalization towards violence, in addition to a lack of trust in the reporting system, could be claimed to be responsible. A considerable percentage did not know how to report, which indicates that they were not oriented towards the availability of a reporting system or were not trained in how to report violence. Many physicians viewed that an apology is enough not to file a complaint. This may be explained by the percentage of physicians who consider violence as a part of the job. This high prevalence of violence with no systematic managerial response may be responsible for the significant number of physicians intending to quit their jobs. Most of them considered leaving the country, which aggravates the problem of Egyptian physicians’ emigration and shortage.

There is a global consensus that WVP should be eliminated and not be tolerated in the healthcare sector, providing a safe environment for HCWs [32, 47]. Considering the significant impact of WPV on HCWs, service quality, and the organizational climate, there is a broad agreement that violence prevention programs should be developed and executed. OSHA has released guidelines to assist healthcare settings in establishing such programs [26]. Among the five elements of OSHA’s preventive program, environmental and administrative control measures should be applied after the risk assessment step [48]. In this study, OSHA’s guidelines’ measures to combat WPV were assessed from the physicians’ point of view. Unfortunately, most of these measures were poorly applied in both hospitals, where the percentage score average was less than 60%; a finding that is not surprising with this high level of WPV detected. Among all measures involving presence and number of security staff were at an acceptable level. Some important environmental measures were hardly present, such as comfortable waiting areas, restricting public access to reception, the ability of physicians to observe patients in the waiting areas, and the presence of possible exits in case violence occurs. Both hospitals had poor organizational-related preventive factors for four out of the five items studied. Staffing and resources were defective, especially in the HIO hospital. There was a critical defect regarding security preventive measures, workplace measures, and organizational support in the MOHP hospital. The training percentage score was zero, a finding that needs to be brought to the surface, and at the same time represents a feasible objective to act upon and improve.

The study showed that there are critical defects in all WPV preventive measures recommended by OSHA, although many studies have shown that violence prevention programs can be effective. In New York, 1996, all psychiatric facilities developed and implemented a proactive violence-prevention program based on guidelines issued by the US Occupational Safety and Health Administration. The impact of the guidelines on worker health and safety in 3 in-patient facilities was evaluated, showing that the staff perception of the quality of management commitment and employee involvement in violence-prevention was significantly improved in all worksites post-implementation [49]. Many studies tried to identify the impact of such programs. Preventive measures studied included WPV policies and procedures such as risk assessment and recordkeeping, environmental factors such as secure entrances/exits with metal detectors and having an efficient alarm system, increasing the risks of getting caught by installing cameras, reducing risk factors as appropriate waiting areas, organizational strategies for WPV prevention such as minimizing the risk of working alone and supportive manager response in follow-up of a violent episode [50–54].

Health authorities should develop and implement clear policies that outline guidelines for preventing, reporting, and managing WPV in healthcare settings, ensuring a standardized approach across all healthcare facilities. A robust system to monitor, track, and investigate WPV incidents across healthcare facilities, allowing for the identification of trends, informed decision-making, and better resource allocation to address the issue effectively, is needed. Organizational support is vital. This involves developing clear policies and procedures for reporting and managing WPV, resource allocations for supporting before, during, and after incidents, promoting a culture of safety and respect, and committing leadership toward preventing WPV. Hospitals should prioritize improvements in workplace preventive procedures, such as companion restrictions and triage redesign for WPV prevention. The national accreditation system (GAHAR) would have a positive effect on the fulfillment of the OSHA guidelines. Comprehensive, tailored training programs on WPV orientation, reporting, and prevention should be obligatory, regularly updated, and adapted to the specific needs of physicians and the hospital environment. 

## Data Availability

The datasets used and/or analyzed during the current study are available from the corresponding author upon reasonable request.

## Abbreviations

%PM: Percentage score of physicians’ perception of the existing preventive measures
EDs: Emergency Departments
GAHAR: General Authority for Healthcare Accreditation and Regulation
HCWs: Healthcare Workers
HIO: Health Insurance Organization
ICU: Intensive Care Unit
MOHP: Ministry of Health and Population
OSHA: The Occupational Safety and Health Administration
SPSS: Statistical Package for Social Sciences
WPV: Workplace Violence
